# Supplementary material for: A structural mechanism for directing corepressor-selective inverse agonism of PPARγ
Source: Nat Commun. 2018 Nov 8;9:4687. doi: 10.1038/s41467-018-07133-w (PMC6224492; doi:10.1038/s41467-018-07133-w)
Supplement: Supplementary file 1 — Supplementary Information [file 41467_2018_7133_MOESM1_ESM.pdf]

Supplementary information for:

A structural mechanism for directing corepressor-selective inverse agonism of PPARγ

R. Brust *et al.*

Supplementary Figure 1. Noncovalent ligands used in the study.

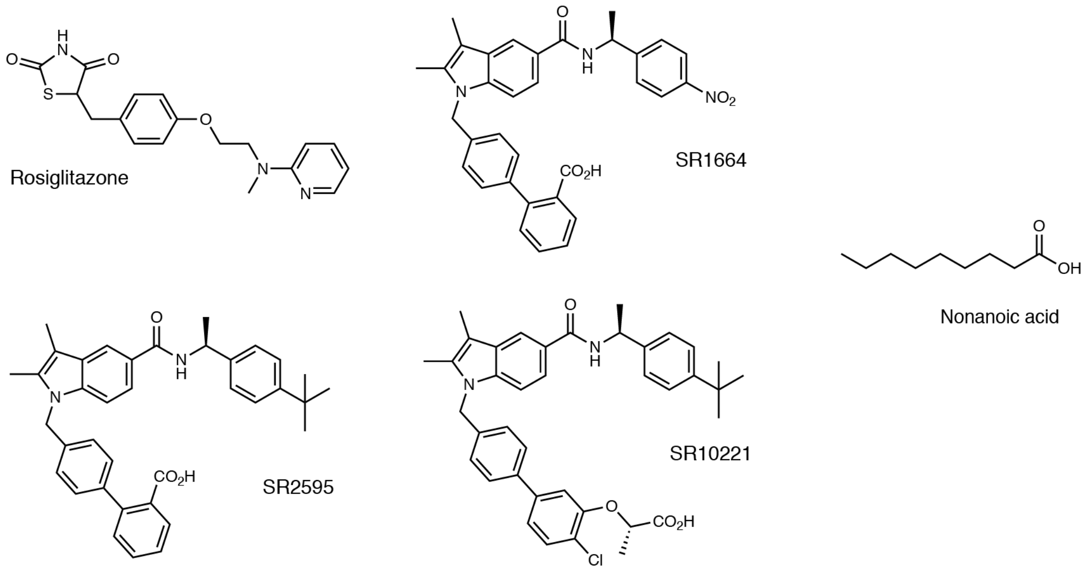

Related to Figure 1.

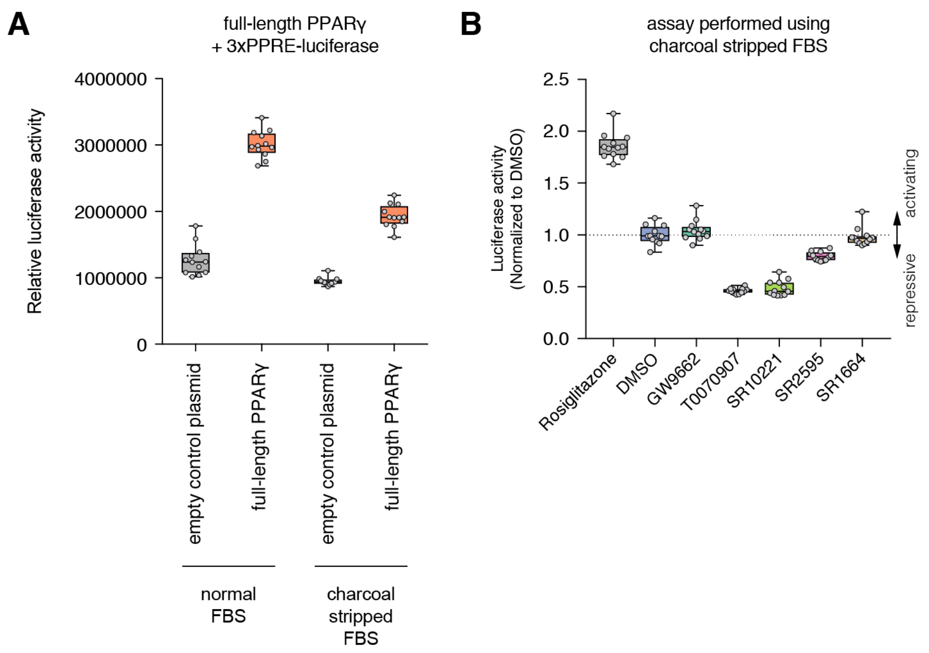

Supplementary Figure 2. Basal cellular activity of full-length PPARγ in a transcriptional reporter assay performed with normal FBS or charcoal stripped FBS shows that exogenous ligands present in FBS cell culture media do not influence synthetic ligand pharmacological properties.

Related to Figure 1. (**A**) HEK293T cells, cultured in normal FBS or charcoal stripped FBS, were transfected with a transcriptional reporter plasmid containing three copies of the PPAR direct repeat 1 (DR1) DNA-response element (PPRE) upstream of the firefly luciferase gene (3xPPRE-luciferase) along with either an empty control plasmid or a full-length PPARγ expression plasmid. Compared to the empty control plasmid, transfection of a full-length PPARγ expression plasmid shows observable transcriptional activation in the absence of added exogenous agonist in cells cultured with normal FBS or charcoal stripped FBS. This observable constitutive activity enables the detection of exogenously added ligand-induced repression of PPARγ (decreased luciferase activity vs. cells transfected with full-length PPARγ and treated with DMSO control) and activation (increased luciferase activity vs. cells transfected with full-length PPARγ and treated with DMSO control) of PPARγ. (**B**) Cell-based full-length PPARγ luciferase transcriptional assay in HEK293T cells cultured in charcoal stripped FBS shows similar activating, neutral, and repressive profiles for the PPARγ ligands (5 μM) profiled in **Figure 1B**. Individual points (n=12), normalized to DMSO control (mean) in (**B**), are plotted as white circles on top of a box-and-whiskers plot; the box represents 25th, median, and 75th percentile of the data, and the whiskers plot the entire range of values. Data are representative of at least 2 independent experiments.

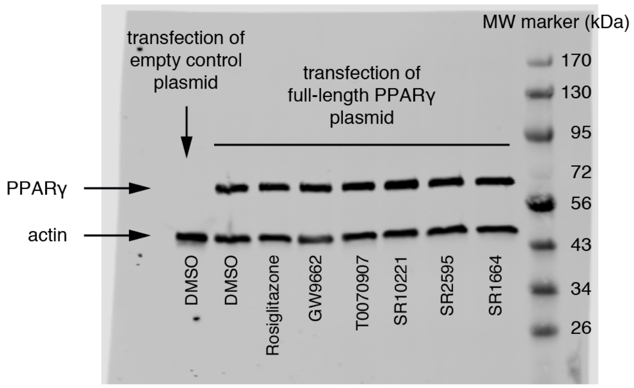

Supplementary Figure 3. Western analysis shows synthetic ligands do not affect PPARγ levels.

Related to Figure 1. The synthetic ligands tested at 5 μM (the same concentration used in the other cellular assays) show no change in the level of PPARγ protein present in HEK293T cells transfected with a full-length PPARγ expression plasmid compared to DMSO control treated cells as detected by Li-COR multiplexed detection western analysis.

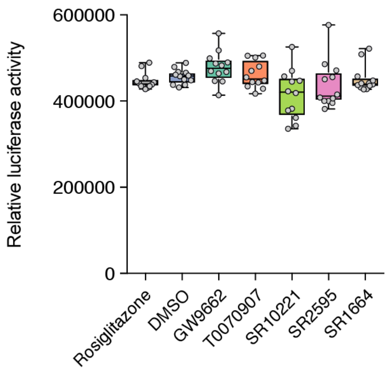

Supplementary Figure 4. The synthetic PPARγ ligands do not cause cytotoxicity at the concentrations tested in the cellular assays.

Related to Figure 1. Using a CellTiter-Glo™ assay, we found that the synthetic ligands tested at 5 μM (the same concentration used in the other cellular assays) show no significant cytotoxicity. Individual points (n=12) are plotted as white circles on top of a box-and-whiskers plot; the box represents 25th, median, and 75th percentile of the data, and the whiskers plot the entire range of values. Data are representative of at least 2 independent experiments.

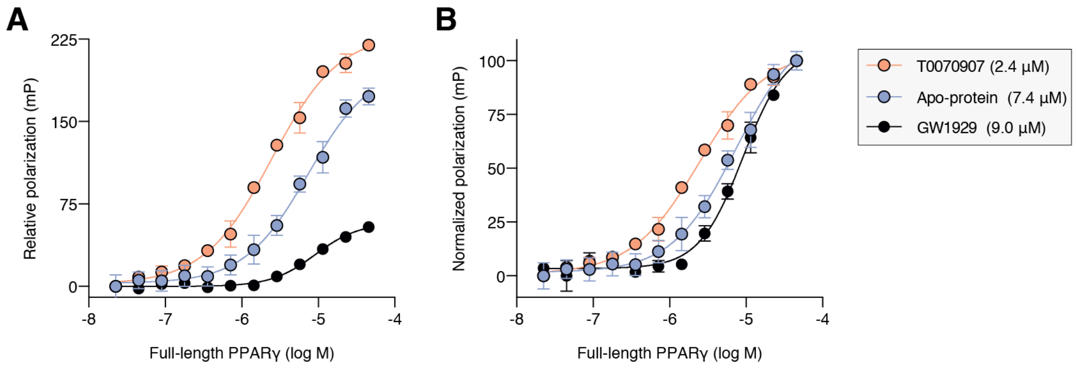

Supplementary Figure 5. Fluorescence polarization assay of NCoR peptide binding to full-length PPARγ.

Related to Figure 2. (**A**) Raw FP polarization values and (**B**) normalized FP polarization values that better show the differences in the binding curves. Fitted K_d values from a one-site binding equation are shown in the legend. Data are representative of at least 2 independent experiments.

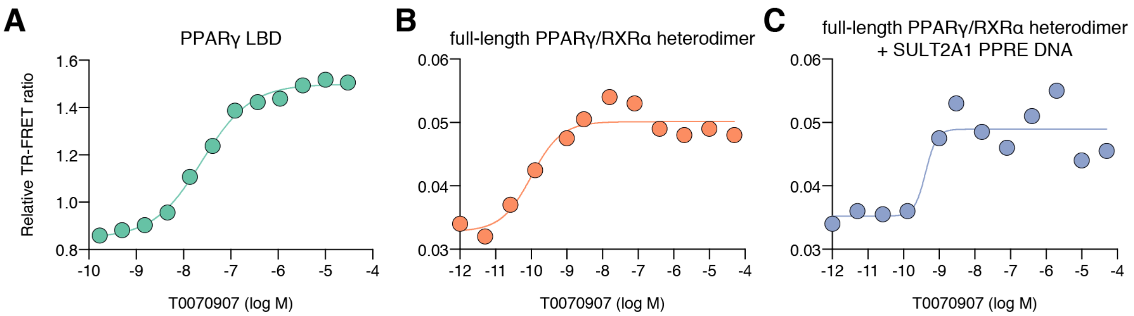

Supplementary Figure 6. Time-resolved fluorescence resonance energy transfer (TR-FRET) assay showing T0070907 increases the interaction of NCoR peptide to PPARγ.

Related to Figure 2. Shown is the effect of T0070907 on the interaction between NCoR peptide and (**A**) PPARγ LBD, (**B**) full-length PPARγ/RXRα heterodimer, and (**C**) full-length PPARγ/RXRα heterodimer bound to a PPAR DNA response element (PPRE) sequence from the *SULT2A1* promoter. The assays show varied *effective* EC_{50} values because T0070907, which covalently binds to PPARγ, was not preincubated with PPARγ protein before the assay. Rather, T0070907 was added to PPARγ protein in the TR-FRET assay plate, which was allowed to incubate ~2 hours before reading. Differences in the amount of incubation time will lead to differences in EC_{50} value and can eventually lead to a steep EC_{50} curve (as in the case of **C**) once all the protein in the assay plate is covalently modified. Furthermore, the assay y-axis window and relative TR-FRET values are different in **A** compared to **B,C** for two reasons. First, the assay in **B,C** use full-length proteins, where the anti-His TR-FRET antibody is much further away from the LBD where the NCoR peptide binds. Second, a different TR-FRET assay modality was used in **A** (FITC-labeled NCoR peptide; fluorophore is directly attached to the peptide) vs. **B,C** (biotin-labeled NCoR peptide + streptavidin-d2 fluorophore). Data are representative of at least 2 independent experiments.

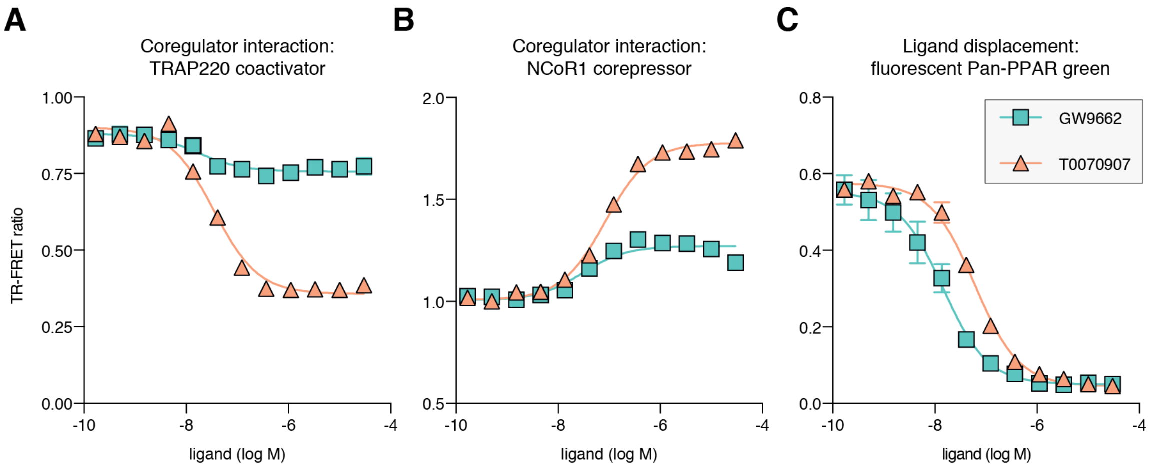

Supplementary Figure 7. TR-FRET direct ligand binding assays show GW9662 and T0070907 have similar relative potencies.

Related to Figure 2. (**A**) TRAP220 coactivator TR-FRET interaction assay (GW9662 IC_{50} = 17 nM; T0070907 IC_{50} = 35 nM). (**B**) NCoR1 corepressor TR-FRET interaction assay (GW9662 IC_{50} = 30 nM; T0070907 IC_{50} = 85 nM). (**C**) Fluorescent Pan-PPAR green TR-FRET ligand displacement assay (GW9662 IC_{50} = 14 nM; T0070907 IC_{50} = 80 nM). Data plotted as the mean (n=2) with error bars representative of s.d. and fit to a sigmoidal dose-response equation to obtain ligand EC_{50}/IC_{50} values. Data are representative of at least 2 independent experiments.

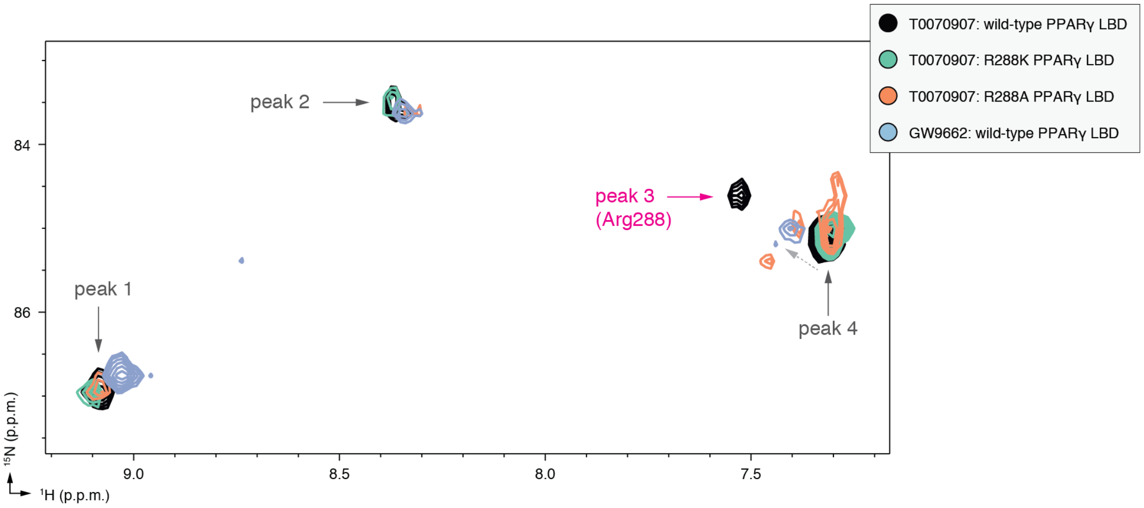

Supplementary Figure 8. Assignment of the R288 Nε-Hε group from 2D [^{1}H,^{15}N]-HSQC NMR spectra of T0070907-bound wild-type PPARγ or R288 mutants.

Related to Figure 3. Spectral overlay focused on the region where arginine side chain Nε-Hε group are found reveals that one (peak 3) out of four observed NMR peaks of T0070907-bound wild-type PPARγ LBD (black peaks, numbered 1–4) disappeared in the R288K and R288A mutants (green and red peaks, respectively), identifying the NMR peak corresponding to the Arg288 Nε-Hε group. The same peak is also absent in GW9662-bound wild-type PPARγ LBD (blue peaks); this is inferred using the minimal NMR chemical shift method[1] whereby the shifted GW9662-bound peak and shifted T0070907-bound R288A peaks (notated with a grey dotted arrow), relative to the wild-type and R288K T0070907-bound peaks, belongs wild-type peak 4. Related to peak 4, the cluster of peaks observed for T0070907-bound R288A but not wild-type or R288K indicates that the pyridyl-water network may be involved in stabilizing the conformational dynamics of this Arg side-chain. *Note*: only 4 out of 8 expected peaks for wild-type PPARγ LBD in the spectral region for Arg Nε-Hε groups is observed. The missing peaks could correspond to Nε-Hε groups with dynamics on the intermediate exchange NMR time scale or solvent exposed groups with rapid exchange with solvent, as both scenarios could result in broadening or absence of expected peaks.

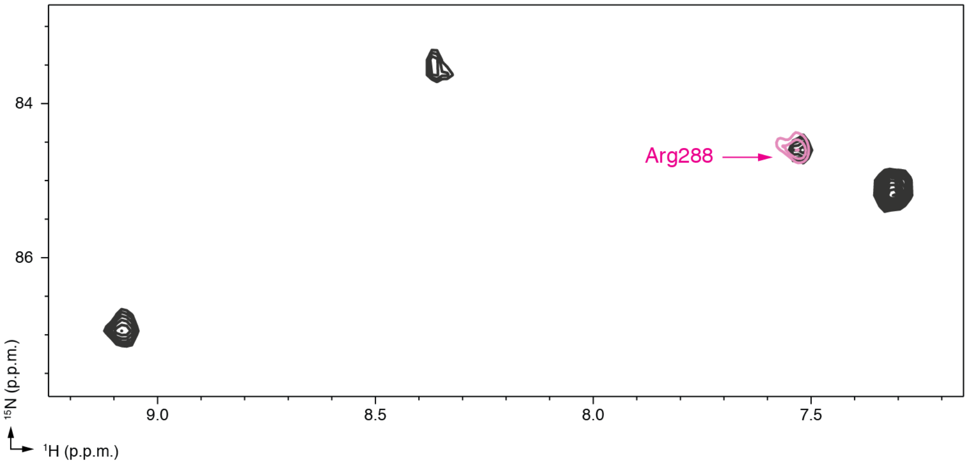

Supplementary Figure 9. CLEANEX-PM NMR analysis confirms the water interaction with the R288 Nε-Hε group.

Related to Figure 3. Spectral overlay of a 2D [^{1}H,^{15}N]-HSQC NMR spectrum (black) and a 2D [^{1}H,^{15}N]-(CLEANEX-PM)-FSQC NMR spectrum (pink; mixing time = 100 ms) of T0070907-bound PPARγ focused on the region where arginine side chain Nε-Hε group. The visible peak in the CLEANEX-PM experiment confirms the 3D ^{15}N-NOESY-HSQC experiment, which revealed a water interaction with the R288 Nε-Hε group.

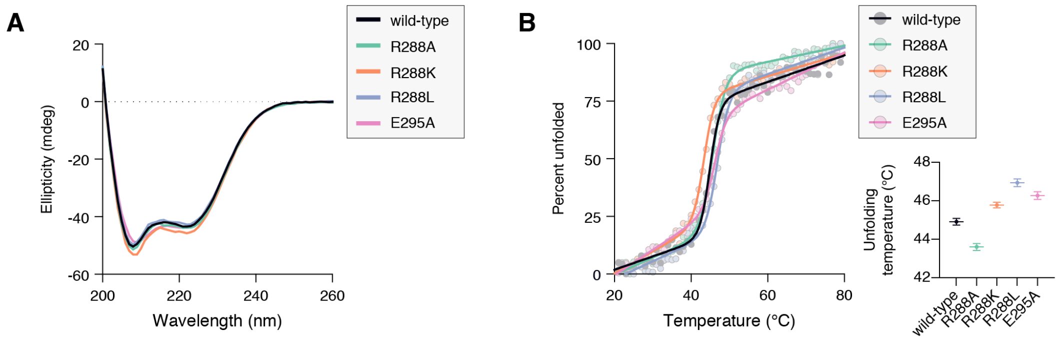

Supplementary Figure 10. Circular dichroism (CD) spectroscopy data on PPARγ LBD mutants.

Related to Figure 4. (**A**) CD spectra and (**B**) CD thermal melt experiments (inset, fitted melting/unfolding temperatures).

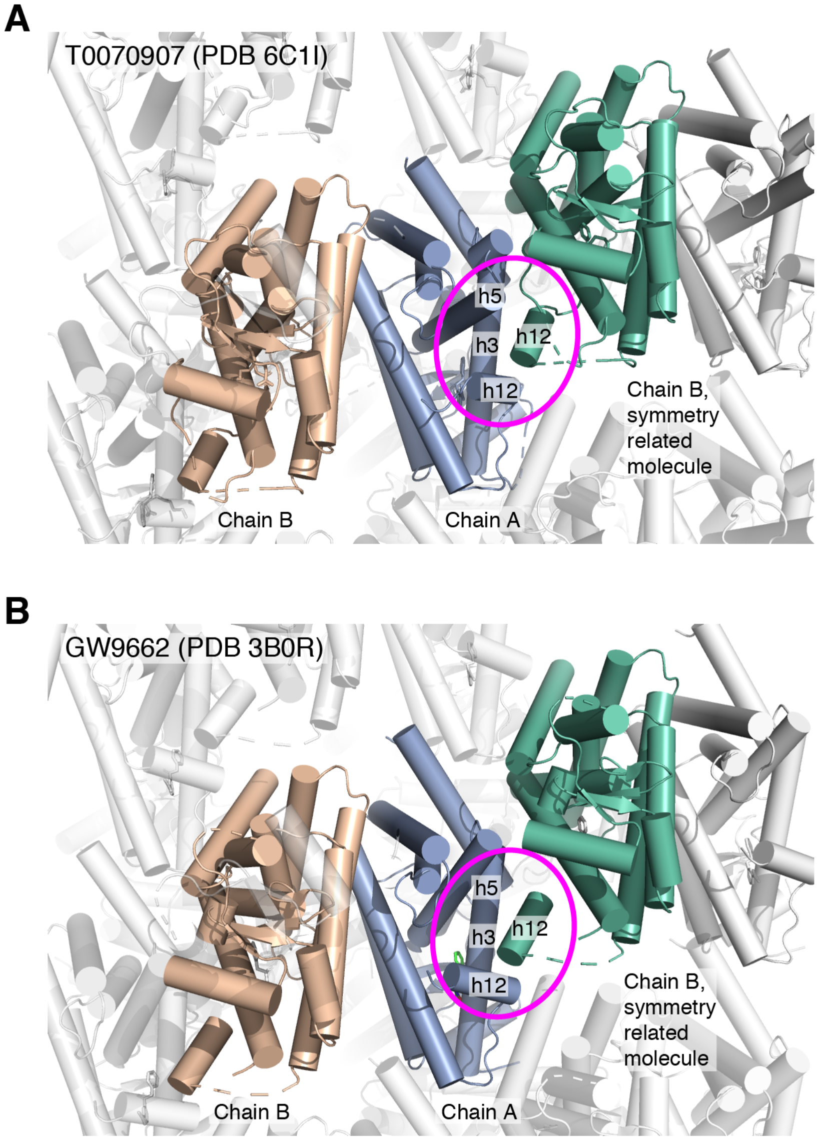

Supplementary Figure 11. The conformation of helix 12 is artefactually influenced due to interchain crystal contacts between different molecules.

Related to Figures 3 and 5. Shown are chain A and B (tan and blue, respectively) and the symmetry related chain B (green) for (**A**) T0070907-bound (PDB 6C1I) and (**B**) GW9662-bound (PDB 3B0R) PPARγ LBD crystal structures. Chain B of helix 12 of the symmetry related molecule, which adopts a conformation commonly referred to as the "inactive" helix 12 conformation, docks into the AF-2 surface (helix 3, 5, and 12) of chain A, artefactually forcing helix 12 in chain A into an active conformation observed in other PPARγ LBD crystal structures where an LXXLL-containing coactivator peptide binds to the same AF-2 site.

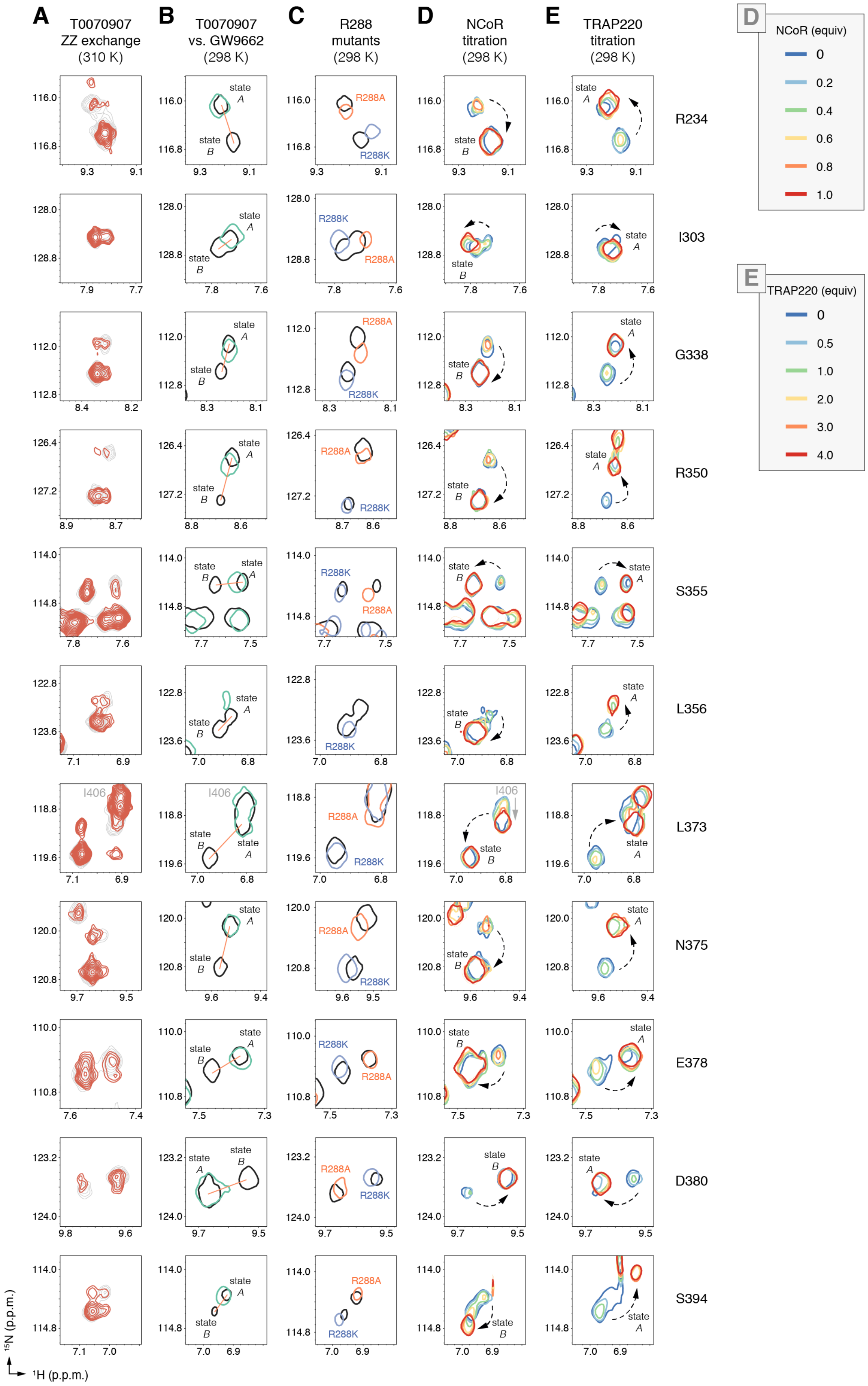
A
T0070907
ZZ exchange
(310 K)
B
T0070907
vs. GW9662
(298 K)
C
R288
mutants
(298 K)
D
NCoR
titration
(298 K)
E
TRAP220
titration
(298 K)
R234
I303
G338
R350
S355
L356
L373
N375
E378
D380
S394
state A
state B
R288A
R288K
I406
D
NCoR (equiv)
0
0.2
0.4
0.6
0.8
1.0
E
TRAP220 (equiv)
0
0.5
1.0
2.0
3.0
4.0
^{15}N (p.p.m.)
^{1}H (p.p.m.)

Supplementary Figure 12. NMR reveals a slow global conformational change between two long-lived conformations with distinct coregulator binding preferences.

Related to Figures 5–8. Data shown for the residues indicated to the right, which are displayed on the PPARγ LBD structure in **Fig. 5B**.

(**A**) Snapshot overlays of ZZ-exchange ^{15}N-HSQC NMR spectra of T0070907-bound ^{15}N-PPARγ LBD; delay = 1 s (red peaks) and 0 s (grey peaks).

(**B**) Snapshot overlays of [^{1}H,^{15}N]-TROSY-HSQC NMR spectra of ^{15}N-PPARγ LBD bound to GW9662 (green) or T0070907 (black) shows that the single GW9662-bound G399 peak has similar chemical shift values to one of the two (connected by an orange line) T0070907-bound G399 peaks (state *A*); state *B* is uniquely populated by T0070907.

(**C**) Snapshot overlays of [^{1}H,^{15}N]-TROSY-HSQC NMR spectra of T0070907-bound ^{15}N-PPARγ LBD wild-type (black), R288K mutant LBD (blue), and R288A mutant LBD (orange). The R288K mutant NMR peak overlaps with the unique wild-type conformation (state *B*), whereas the R288A mutant NMR peak overlaps with the GW9662-bound mutual conformation (state *A*).

(**D,E**) Snapshots of [^{1}H,^{15}N]-TROSY-HSQC spectra of ^{15}N-PPARγ LBD bound to T0070907 and titrated with (**C**) NCoR or (**D**) TRAP220 peptide.

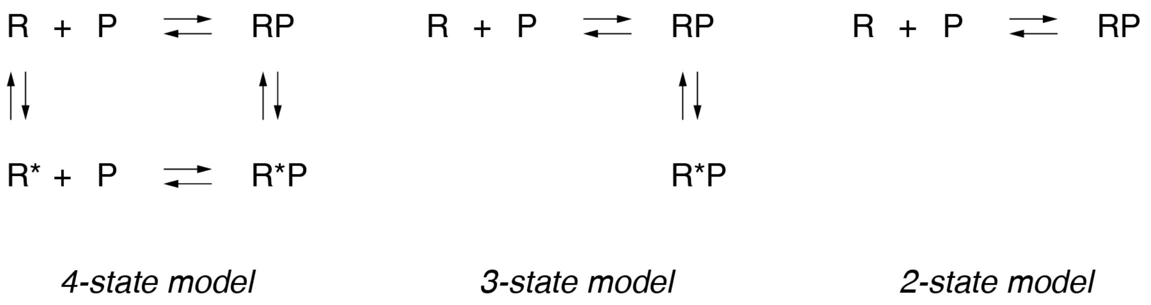

Supplementary Figure 13. Chemical reaction pathways describing the NMR coregulator titration data.

Related to Figures 7 and 8. Models are described in Kovrigin (2012), which are used in the program LineShapeKin (http://lineshapekin.net)

Kovrigin, E.L. (2012). NMR line shapes and multi-state binding equilibria. J Biomol NMR *53*, 257-270.

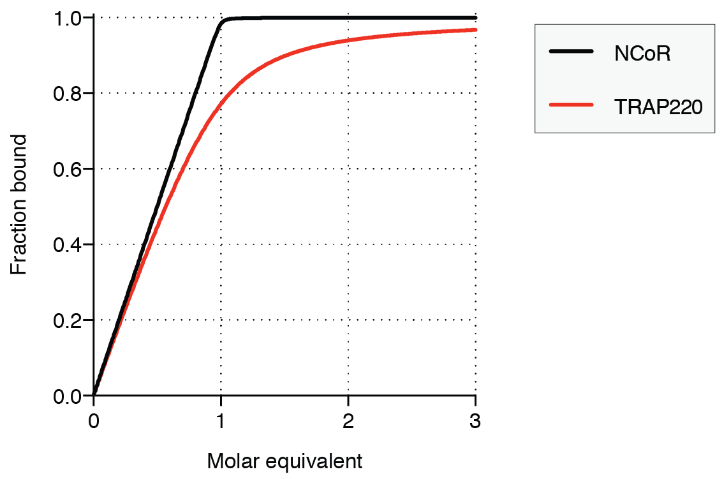

Supplementary Figure 14. Calculation of coregulator peptide-bound fractions in the NMR titration experiments from the peptide K_d.

Related to Figures 7 and 8. Given that the exchange rate measured in the ZZ-exchange NMR analysis of T0070907-bound PPARγ LBD between the two states, *A* and *B*, is relatively fast (seconds) compared NMR data acquisition (hours), the populations should be in equilibrium for all NMR titration data. Thus, the bound NCoR- and TRAP220-bound fractions can be calculated from the K_d values measured from the fluorescence polarization coregulator profiling assay (**Figure 1**) using **Supplementary Equation 1** (see below), which shows similarities to the NMR observed binding trends.

Supplementary Table 1. Pharmacological phenotypes and functions of PPARγ ligands.

**Transcriptional phenotype**	**Properties**
Agonist (full or partial)	Strengthens coactivator binding<br>Weakens corepressor binding<br>Increases transcription
Neutral antagonist	Relatively no change in coregulator binding<br>Relatively no change in transcriptional activity vs. basal activity
Inverse agonist<br>(coregulator inhibitory)	Weakens coactivator binding<br>Weakens corepressor binding<br>Decreases transcription
Inverse agonist<br>(corepressor selective)	Weakens coactivator binding<br>Strengthens corepressor binding<br>Decreases transcription

Supplementary Table 2. DNA primer sequences (5′ to 3′) used for site-directed mutagenesis of PPARγ in this study.

Mutant	Forward Primer	Reverse Primer
E295A	GAGGCTGTGCAGGCGATCACAGAGTATG	CATACTCTGTGATCGCCTGCACAGCCTC
R288A	TTTCAGGGCTGCCAGTTTGCCTCCGTGGAG	CTCCACGGAGGCAAACTGGCAGCCCTGAAA
R288K	GCTGCCAGTTTAAATCCGTGGAGGC	GCCTCCACGGATTTAAACTGGCAGC
R288L	CTGCCAGTTTTTATCCGTGGAGGC	GCCTCCACGGATAAAAACTGGCAG

Supplementary Table 3. DNA primer sequences (5′ to 3′) used for QPCR in this study.

Gene	Forward Primer	Reverse Primer
aP2/FABP4	AAGGTGAAGAGCATCATAACCCT	TCACGCCTTTCATAACACATTCC
CD36	AAGCTATTGCGACATGATT	GATCCGAACACAGCGTAGAT
TBP	AAGGGAGAATCATGGACCAG	CCGTAAGGCATCATTGGACT

Supplementary Equation 1.

f(x) = Rt*Lt-(x*(Lt+Rt+Kd))+(x*x))/Kd

where

Rt = total receptor/protein concentration (200 μM)

Lt = total ligand concentration

Kd = peptide binding affinity (TRAP220 = 13.66 μM; NCoR = 0.06 μM)

x = fraction bound

Supplementary References

1. Williamson, M.P. Using chemical shift perturbation to characterise ligand binding. *Prog Nucl Magn Reson Spectrosc* **73**, 1-16 (2013).
